# Supplementary material for: Atomistic Insights into the Chain-Length-Dependent Antifreeze Activity of Oligoprolines
Source: Biomacromolecules. 2025 Jul 29;26(8):4886–97. doi: 10.1021/acs.biomac.5c00324 (PMC12344715; doi:10.1021/acs.biomac.5c00324)
Supplement: Supplementary file 1 [file bm5c00324_si_001.pdf]

Supporting Information

Atomistic Insights into the Chain-Length-Dependent Antifreeze Activity of Oligoprolines

*Wentao Yang, Yucong Liao, Zhaoru Sun**

School of Physical Science and Technology, ShanghaiTech University, Shanghai 201210, China

* Corresponding Author

E-mail address: sunzhr@shanghaitech.edu.cn (Z.S.)

Table of Contents

Figure S1. Schematic representation of the simulation system used to study ice growth inhibition.

Figure S2. Embedded states of oligoproline molecules on the ice surface.

Figure S3. Representative snapshots of P3, P8L, and P15L being engulfed by ice.

Figure S4. Gibbs free energy profile as a function of coordination number (CN) at low concentration (~20 mg/mL).

Figure S5. Representative metastable configurations of P8 and P15 obtained from metadynamics simulations at higher concentration (~40 mg/mL).

Figure S6. Total number of intermolecular hydrogen bonds formed between P15 molecules as a function of CN at high concentration (~40 mg/mL).

Figure S7. Temperature-dependent aggregation behavior of oligoproline at 40 mg/mL.

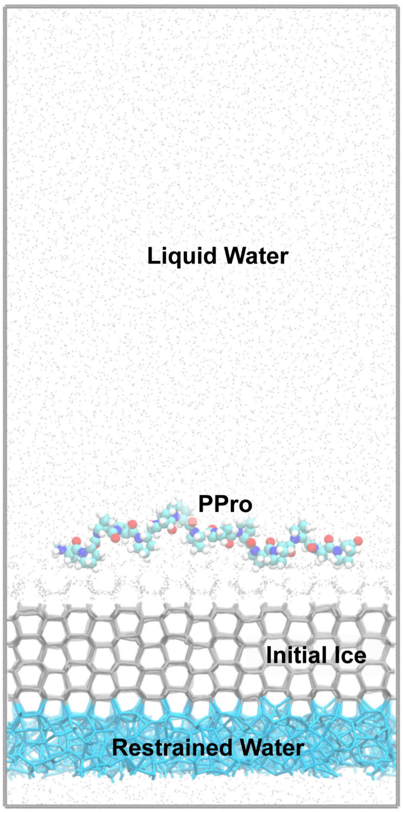

Figure S1. Schematic representation of the simulation system used to study ice growth inhibition. Four layers of initial ice (colored gray) are restrained by a harmonic potential, with the primary prismatic face oriented toward the water phases. The oxygen atoms of liquid water molecules are represented as gray points. A 1 nm thick water layer (colored cyan) below the hexagonal ice slab is restrained to prevent downward ice growth.

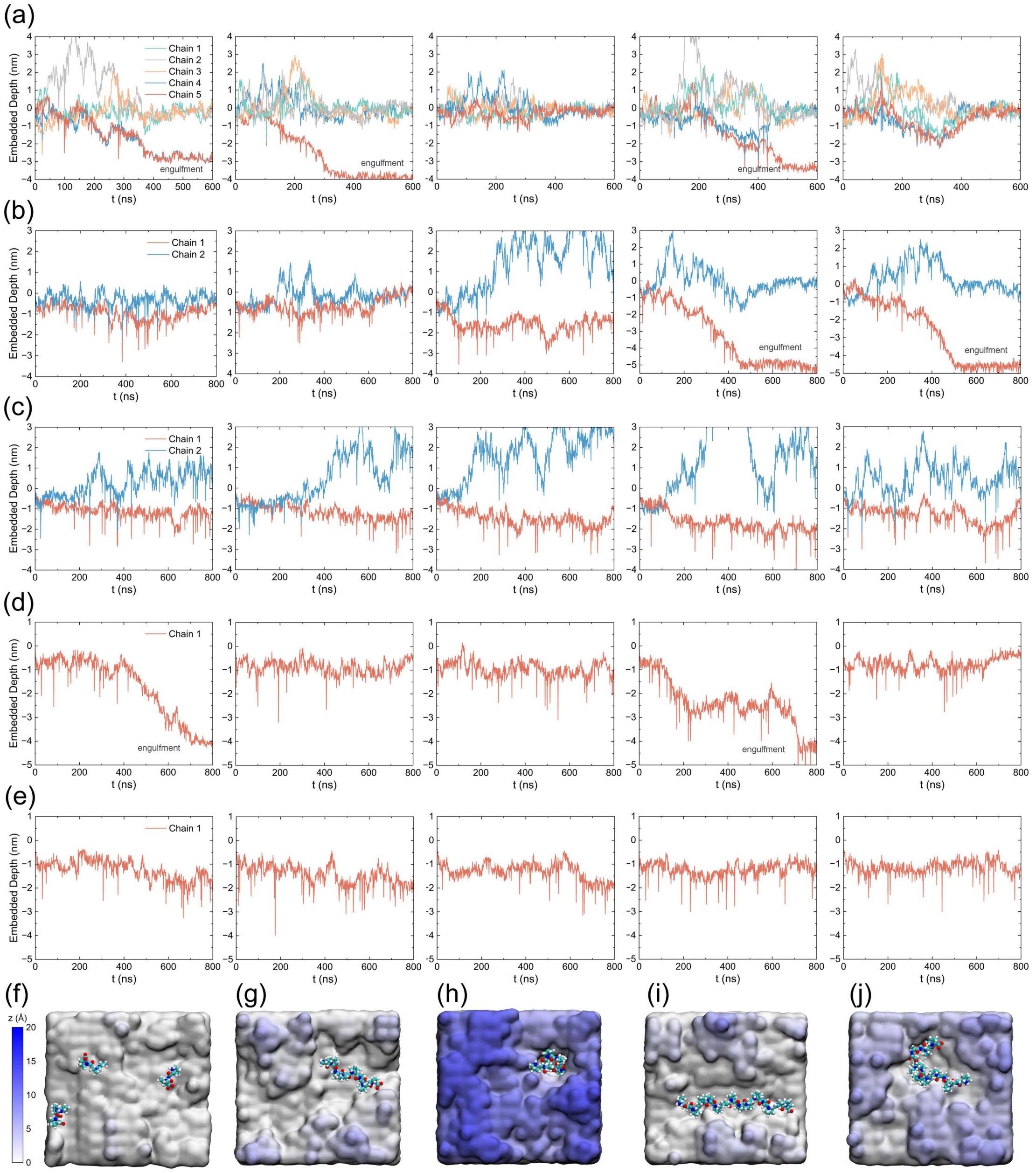

Figure S2. Embedded states of oligoproline molecules on the ice surface. (a-e) Embedded depth (z_{PPro}− z_{ice}) of P3 (a), P8L (b), P8C (c), P15L (d), and P15C (e) at the ice surface. (f-j) Representative binding states and configurations of P3 (f), P8L (g), P8C (h), P15L (i), and P15C (j), with the ice surface color-coded by the z-axis height. The bottom atom of each oligoproline molecule is used as the reference point (z=0). oligoproline molecules that stay away from the ice surface (unbound) are not shown.

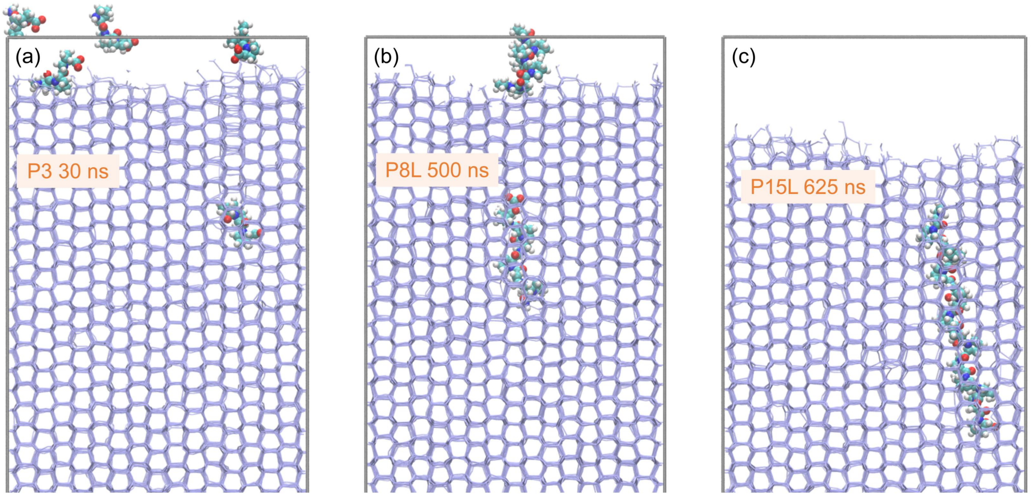

Figure S3. Representative snapshots of P3, P8L, and P15L being engulfed by ice. (a) P3 is too small and is easily engulfed by the growing ice front. (b) P8 and (c) P15 become engulfed by ice when aligned parallel to the growing ice front.

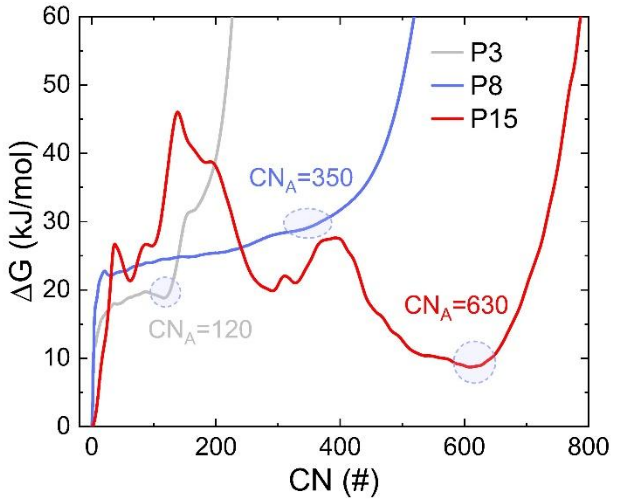

Figure S4. Gibbs free energy profile as a function of coordination number (CN) at low concentration (~20 mg/mL), with very small uncertainty values (less than 0.3 kJ/mol) obtained from our metadynamics simulations.

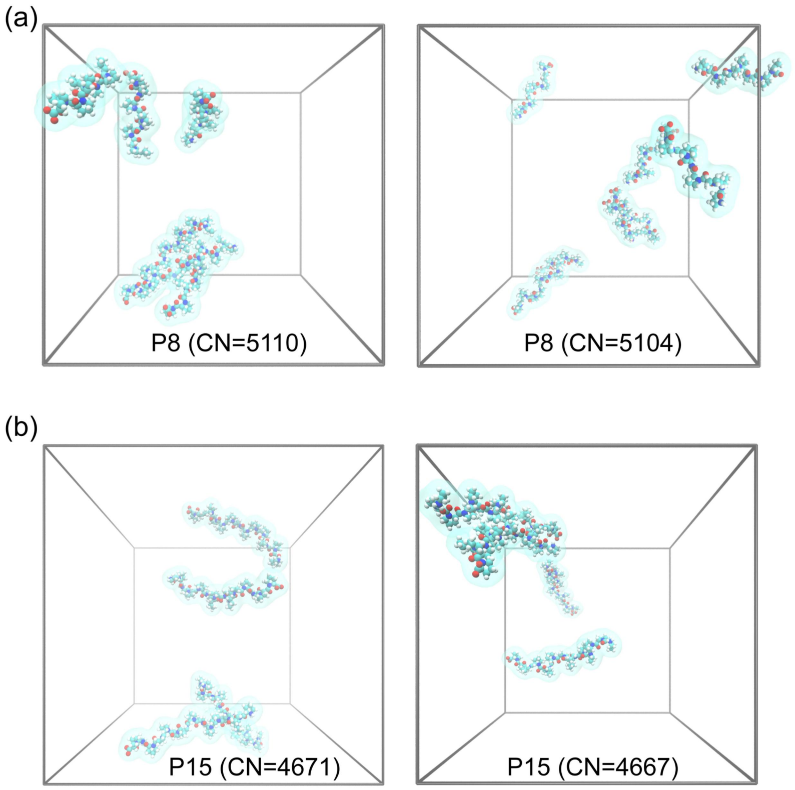

Figure S5. Representative metastable configurations of P8 (a) and P15 (b) obtained from metadynamics simulations at higher concentration (~40 mg/mL). These states primarily involve unstable dimer formations.

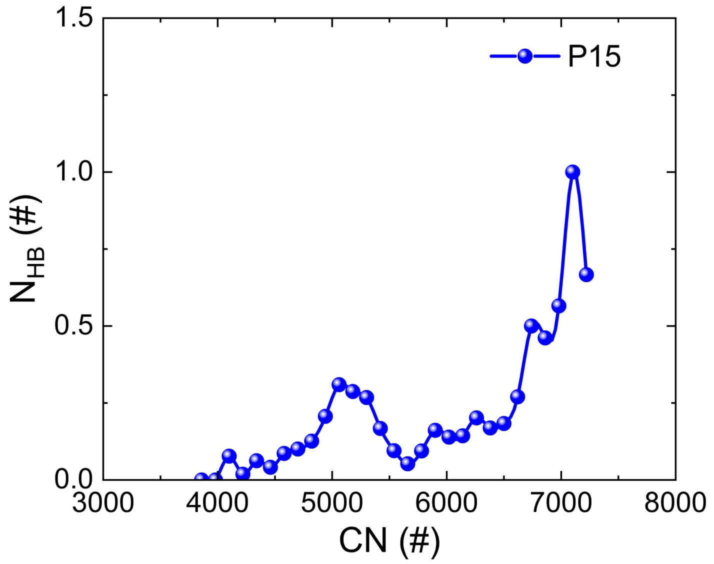

Figure S6. Total number of intermolecular hydrogen bonds formed between P15 molecules as a function of CN at high concentration (~40 mg/mL). The results show that almost no intermolecular hydrogen bonds are formed within P15 aggregations.

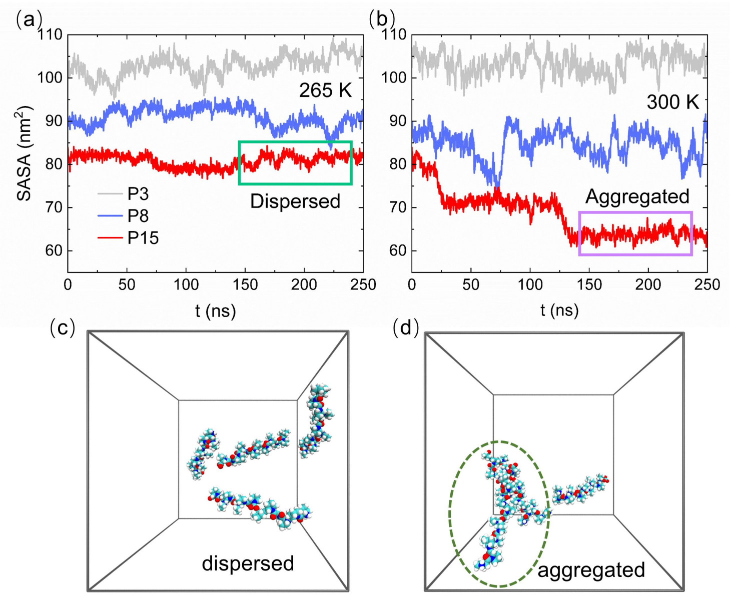

Figure S7. Temperature-dependent aggregation behavior of oligoproline at 40 mg/mL. (a) Time evolution of solvent accessible surface area (SASA) for P3, P8, and P15 at 265 K, indicating that all oligoproline chains remained in the dispersed state. (b) Time evolution of SASA for P3, P8, and P15 at 300 K. The result suggests that P15 tends to aggregated states, while P3 and P8 remain in dispersed states. (c) Representative configuration of P15 in the dispersed state (SASA ≈ 80 nm^2). (d) Representative configuration of P15 in the (partially) aggregated state (SASA ≈ 65 nm^2)
